# Effective programs for bovine embryo transfer in *Bos taurus* and *Bos indicus* cattle

**DOI:** 10.1590/1984-3143-AR2026-0068

**Published:** 2026-08-28

**Authors:** Gabriel A. Bó, Andrés Vera Cedeño

**Affiliations:** 1 Instituto de Reproducción Animal Córdoba – IRAC, Córdoba, Argentina; 2 Instituto de A.P. de Ciencias Básicas y Aplicadas, Medicina Veterinaria, Universidad Nacional de Villa María, Villa del Rosario, Córdoba, Argentina; 3 Instituto de Reproducción Animal del Ecuador – IRAE, Guayaquil, Ecuador

**Keywords:** estradiol, GnRH, estrus, pregnancy, pregnancy losses

## Abstract

The main objective of implementing embryo transfer programs in beef operations is to accelerate the rate of genetic progress in the herd. The main factors that affect the use of this technology are related to nutrition, management, category and breed of the recipients and the estrus synchronization programs implemented. As a result of research conducted over the last 30 years, recipient utilization has increased by applying protocols that synchronize ovulation and allow for embryo transfer without the need for estrus detection, usually referred to as fixed-time embryo transfer (FTET). Although these protocols have performed adequately for several years, recent attention has been directed to the effect of estrus expression, proestrus length and restriction to some hormonal treatments in some countries. The experiments reviewed herein demonstrate that several programs can be effectively used today to obtain high pregnancy per ET (P/ET) and reduced pregnancy losses, especially in recipients receiving in-vitro-produced embryos.

## Introduction

The main objective of an embryo transfer program is to increase the genetic value of the offspring produced in a herd. Nutrition, management and efficiency in the detection of estrus are among the factors that affect the use of this technology (Mapletoft and Bó, 2016). The protocols that synchronize estrus and ovulation, usually referred to as fixed-time embryo transfer (FTET), have allowed for the widespread application of the embryo transfer technology around the world (Bó et al., 2002, 2012a). The objective of this manuscript is to briefly review protocols that are used to synchronize ovulation and discuss how they impact on the application of commercial embryo transfer programs in *Bos taurus* and *Bos indicus* cattle.

## Conventional synchronization treatments for embryo recipients in South America

Although prostaglandin F_2α_ (PGF_2α_) alone was used for many years for synchronization of estrus in recipients, the requirement for estrus detection and the variability in the interval from treatment to estrus and ovulation adversely affected its performance in embryo transfer programs, especially in *Bos indicus* cattle (reviewed in Bó et al., 2002).

Due to the difficulties in estrus detection, most of the recipient programs used in South America are treatments that synchronize the time of ovulation, which were developed originally for fixed-time AI (FTAI). These treatments are generally divided into those that are GnRH-based and those that are estradiol (E2)-based (Bó et al., 2002). In either case, the recipient protocols include the insertion of a progesterone (P4) releasing device for 7 or 8 d (Hinshaw 1999; Bó et al., 2002; Sala et al., 2020).

The E2/P4-based protocol used most commonly nowadays consists of insertion of a P4-releasing device and the administration of 2 mg estradiol benzoate (EB) on day 0, and PGF_2α_ at the time of insertion and removal of the P4 device if it is impregnated with >1g of P4 (in *Bos indicus* recipients) and only at P4 device removal when it contains <1 g of P4 and in *Bos taurus* recipients. The P4 device is usually removed on day 7 or 8 and 300 to 400 IU of equine Chorionic Gonadotropin (eCG) is given at that time (Baruselli et al., 2010). Ovulation is induced by the administration of 0.5 or 1 mg of estradiol cypionate (ECP) at the time of P4 device removal and all recipients with a CL receive an embryo 9 d later (i.e., 7 d after the expected time of estrus; Baruselli et al., 2010, 2011; Bó et al., 2012a, b).

Overall, 75 to 85% of the recipients treated with this protocol receive an embryo (defined as utilization rate), compared to 50% or less with PGF_2α_ synchronization. Furthermore, P4 concentrations are high at the time of embryo transfer and P/ET usually exceeds 50%, when both embryos and recipients are of high quality (reviewed in Bó et al., 2002; Baruselli et al., 2010, 2011).

## Expression of estrus, preovulatory estradiol and the establishment of pregnancy

It has been suggested that the progression of events required for conceptus growth, elongation, survival and attachment are influenced by the coordination of events leading to a decrease in P4 concentrations and an increase in E2 concentrations prior to the onset of estrus (Bridges et al., 2013). Additionally, preovulatory E2 concentrations have been reported to have a positive impact on subsequent conceptus development, and cows that exhibit estrus have been reported to have a greater conceptus length on day 19 of gestation compared to those not exhibiting estrus (Davoodi et al., 2016).

The occurrence of estrus in FTAI programs has been shown to be positively associated with pregnancy per AI (P/AI) in *Bos taurus* (Richardson et al., 2016), *Bos taurus x Bos Indicus* (Cedeño et al., 2021) and *Bos indicus* beef cattle (Sá et al., 2011). Similarly, the expression of estrus also has been shown to have a significant effect in P/ET and pregnancy losses in recipients synchronized with FTET protocols (Cedeño et al., 2020). In GnRH-based protocols the reason for the higher P/ET in recipients showing estrus is that they were exposed to higher E2 concentrations than those that were induced to ovulate with GnRH prior to showing estrus and the higher P4 concentrations at the time of FTET. In the study performed by Atkins et al. (2013), single embryos (n=354) that were obtained from cows that were induced to ovulate a large or a small follicle with GnRH were transferred into recipients that were also induced to ovulate a small or a large follicle with GnRH. Pregnancy maintenance from 7 to 27 d of gestation was enhanced by increased serum E2 concentrations at the time of the GnRH treatment and P4 concentration 7 d later in the recipient cows. However, this study also showed that follicle diameter was not all that important, as recipients with large follicles had the lowest P/ET, indicating that E2 produced by a new growing dominant follicle will benefit pregnancy more than an aged large dominant follicle that has already reduced E2 production at the time of GnRH-induced ovulation (Bridges et al., 2014). These results were also confirmed in two studies in which E2 treatments were administered to increase circulating E2 concentrations prior to the induction of ovulation in cows synchronized with GnRH-based protocols (Jinks et al., 2013; Madsen et al., 2015).

The effects of E2 concentrations and estrus expression on P/ET in recipients treated with E2/P4-based protocols has also been reviewed. A retrospective analysis of several experiments performed on commercial dairy farms in Brazil revealed a positive association between estrus expression and fertility (Pereira et al., 2016). Pereira et al. (2016) evaluated lactating dairy cows that were either artificially inseminated (n=5430) or used as recipients (n=2003). All cows were treated with a CIDR (Zoetis, Brazil) and 2 mg EB on day 0, and PGF_2α_ on day 7, and CIDR removal and 1 mg of ECP on day 9. Cows were either FTAI on day 11 or FTET with *in vitro* produced (IVP) embryos on day 18. Estrus was detected using tail-head devices and pregnancy was determined on days 32 and 60 of gestation. Estrus expression positively influenced (P<0.01) P/AI on Day 32 of gestation (estrus: 38.9% vs no estrus: 25.5%) and P/ET (estrus: 46.2% vs no estrus: 32.7%). Furthermore, pregnancy loss to 60 d was lower (P<0.01) in cows that expressed estrus in FTAI (estrus: 14.4%, vs no estrus: 20.1%) and FTET (estrus: 18.6% vs no estrus: 22.7%). Similar results were also reported with crossbred *Bos indicus x Bos taurus* beef heifers; the manifestation of estrus behavior up to 3 d after P4 device removal increased P/ET in recipients (n=170) receiving IVP embryos (Frade et al., 2014). Heifers expressing standing estrus had greater P/ET (62.4%) than heifers that did not express estrus (47.0%; P<0.01). In addition, heifers that became pregnant had greater circulating P4 concentrations at FTET (2.8±0.14 ng/mL; n = 137) than those that did not become pregnant (2.2±0.18 ng/mL; n= 99; P = 0.04; Frade et al., 2014). Thus, the sequential exposure to greater concentrations of E2 during the pre-ovulatory phase and the subsequent exposure to high P4 in the diestrus positively influenced pregnancy success after FTET.

Two other studies were performed in Argentina to confirm that the expression of estrus had a positive effect on P/ET and maintenance of pregnancy in recipients treated with E2/P4-based protocols (Cedeño et al., 2021). A secondary objective was to evaluate if the administration of GnRH at the expected time of estrus to recipients not showing estrus would increase the proportion of recipients transferred and pregnant. In the first experiment, beef cows (n=729) were treated with 2 mg EB and an intravaginal P4 device containing 0.5 g of P4 (DIB 0,5, Zoetis). Devices were removed 8 d later and all cows received PGF2α, 400 IU eCG, and 0.5 mg ECP at that time. Expression of estrus was determined at 48 and 56 h after device removal using tail-paint and cows that did not show positive signs of estrus by 48 h received GnRH or no treatment at random. The overall estrus rate was 76.0% (554/729); 68.0% had positive signs of estrus by 48 h after P4 device removal and 28.0% of those not in estrus by 48 h showed estrus by 56 h. The proportion of recipients receiving *in vivo*-derived (IVD) or IVP embryos and P/ET were greater in recipients that showed estrus by 48 and 56 h (94.0% and 48.4%, respectively) than in those that did not show estrus (41.0% and 29.0%, respectively; P < 0.01). However, GnRH treatment of recipients not showing estrus by 48 h did not improve P/ET. Experiment 2 evaluated the effect of expression of estrus on P/ET and pregnancy loses up to parturition in recipients synchronized with two E2/P4-based protocols. Beef *Bos indicus x Bos taurus* cows (n=403) were divided at random to receive the same synchronization protocol as in Experiment 1 (ECP) or a J-Synch protocol (device removal on day 6 and without using ECP to induce ovulation). In this experiment, pregnancy was determined at 30 and 60 d by ultrasonography, and all pregnant recipients were followed until parturition to determine pregnancy losses during gestation. Although the number of recipients receiving IVP embryos was greater in the ECP group (90.5% vs. 83.5%; P<0.05), P/ET on day 30 of gestation did not differ among treatment groups (ECP: 37.0% and J-Synch: 39.0%; P = 0.43). Overall, 88.0% (357/407) of the recipients synchronized showed estrus and a greater P/ET (P<0.05) was found in the recipients that showed estrus (39.0%) vs. those that did not show estrus (26.0%), regardless of treatment group. Pregnancy losses were lower (P<0.01) and the calving rate was greater (P<0.01) in recipients that showed estrus (25.0% and 29.3%, respectively) than in those that did not (88.8% and 2.9%, respectively). In summary, expression of estrus was associated with a greater P/ET in recipients treated with two E2/P4-based synchronization protocols. The expression of estrus was associated with a greater proportion of recipients receiving embryos, P/ET and calving rate. Treatment with GnRH did not improve P/ET in the recipients that did not show estrus, questioning its use in recipients synchronized with E2/P4 based FTET protocols. The potential practical drawback of these results was that doing embryo transfer in only recipients that express estrus would reduce the utilization rate of the recipients synchronized, which has been shown as one of the main benefits of using FTET programs in recipients in South America (Bó et al., 2002, 2012b; Baruselli et al., 2010, 2011). However, the pregnancy losses are less, which is also very important for the producers and the widespread application of embryo transfer technology, specially using IVP embryos.

## Protocols that prolong the proestrus period

Studies performed originally in United States using the GnRH-based protocol named 5-day Co-Synch+P4 have suggested that increasing the interval from P4 device removal to GnRH improves P/AI as compared to the traditional 7-day GnRH+P4 protocol in beef cattle (Bridges et al., 2008). Furthermore, using a modified 5-day Co-Synch+P4 protocol (no GnRH at P4 device insertion, PGF_2α_ at P4 removal on day 5 and GnRH on day 8), Sala et al. (2020) reported similar P/ET with IVP embryos to those recipients synchronized with two PGF_2α_ treatments 14 d apart and estrus detection. Based on these findings, we evaluated the effectiveness of an E2/P4 treatment protocol in which the proestrus was lengthened by the administration of GnRH 72 h after P4 device removal instead of ECP at device removal. The protocol for FTAI was named J-Synch (de la Mata and Bó, 2012). This treatment protocol has resulted in greater P/AI in beef heifers compared to the conventional protocol in which the P4 device is removed on day 7 and ECP is given at that time (Bó et al., 2016; de la Mata et al., 2018).

A series of 4 subsequent experiments evaluated the performance of the J-Synch protocol in embryo transfer programs (Menchaca et al., 2016). The experiments involved 3,782 cycling *Bos taurus* beef recipients that received IVP Holstein embryos. The first experiment compared P/ET obtained with the J-Synch protocol to the conventional E2/P4 protocol for FTET. All recipients received a P4 device plus 2 mg EB on day 0. In the J-Synch group (n=464), the P4 device was removed on day 6 and PGF_2α_ and 400 IU eCG were given at the same time. GnRH was administered 72 h later. In the conventional treatment (n=481), the P4 device was removed on day 7 and PGF_2α_, eCG, and 0.5 mg ECP were administered at the same time. In this first study and in the subsequent studies, FTET was performed on days 16 and 17 and P/ET was determined by ultrasonography 40-50 d later. In this study, P/ET was greater in the J-Synch (49.4%, 229/464) than in the conventional E2/P4 synchronization protocol (41.0%, 197/481; P<0.05). The second experiment compared GnRH vs. EB to induce ovulation in recipients synchronized with the J-Synch protocol. The J-Synch protocol was performed as described previously, with GnRH given at 72 h (n=456) or 1 mg EB given at 60 h after P4-device removal (n=461). P/ET did not differ, regardless of whether GnRH was administered at 72 h (58.8%, 230/391) or EB was administered at 60 h (54.7%, 227/415). In Experiment 3, we evaluated the effect of the time of GnRH administration in the J-Synch protocol (GnRH was given at 60 h (n=452) or 72 h (n=466) after P4 device removal) and P/ET was not affected when GnRH was administered at 60 h (47.8%, 216/452) or 72 h (50.4%, 235/466). In the fourth study, cows were treated with the J-Synch protocol and were assigned at random to receive GnRH 72 h after P4 device removed or were allowed to ovulate spontaneously, without using GnRH. It is noteworthy that P/ET was greater for recipients that did not receive GnRH (57.5%, 306/532) compared to those that received GnRH (51.5%, 299/581; P<0.05). The overall conclusion of this sequence of experiments was that the exposure to endogenous E2 prior to ovulation, as it occurs with the prolonged proestrus of the J-Synch protocol, improved P/ET with IVP embryos. Furthermore, results of the fourth experiment suggested that shortening the growth period of the ovulatory follicles with GnRH may adversely affect the chances of pregnancy in some recipient cows. Again, the expression of estrus had a positive effect on P/ET as it was shown in the studies described previously.

## Application of J-Synch protocols in *Bos indicus* recipients

Most of the embryo recipients used in tropical climates are *Bos indicus*, due to their adaptation to this environment. Therefore, an experiment was designed to evaluate P/ET and pregnancy losses in beef recipients synchronized with the J-Synch (i.e. long proestrus) and the conventional E2/P4-based protocol (i.e. short proestrus; Cedeño and Bó, 2021). The experiment was performed in 6 replicates, and all recipients were multiparous non-lactating *Bos indicus* beef cows (n=750) with a CL or a follicle of at least 8 mm in diameter and a body condition score (BCS) between 2.5 and 4 (1 to 5 scale). On day 0, all cows received 2 mg EB and a P4 device and were randomly divided into three treatment groups. The P4 device was removed on day 6 in the J-Synch 6 d group and on Day 7 in the J-Synch 7 d and conventional E2/P4 treatment group (named ECP). All cows received PGF_2α_ and 400 IU of eCG at the time of P4 device removal, and cows in the ECP group also received 1 mg ECP at the same time. All cows had their tails painted to detect estrus. Cows with >30% tail paint removed by 72 h (J-Synch groups) or 48 h (ECP group) after device removal were considered in estrus. Only cows showing estrus were examined by ultrasonography 7 d later and those with a CL >16 mm in diameter were transferred with IVP embryos. Pregnancy was determined at 30 and 60 d by ultrasonography, and all pregnant recipients were followed until parturition to determine pregnancy losses during gestation. The results of this study are shown in Table 1. The proportion of synchronized cows that were transferred was greater (P=0.03) in those in the ECP group than in the J-Synch 6 d and J-Synch 7 d groups. However, P/ET at 30 and 90 d did not differ (P>0.05) between J-Synch 7 d (40.0% and 37%), J-Synch 6 d (42.8% and 38.9%) and ECP (41.8% and 35%) groups. When pregnancy losses from 30 to 90 d was analyzed, this was greater (P=0.04) in the cows in the ECP group (16.1%) than in the J-Synch groups (J-Synch 7 d: 9.0% and J-Synch 6 d: 7.0%). In a similar way, pregnancy losses from 90 d to delivery were greater (P<0.05) in cows in the ECP group (15.3%) than those in the J-Synch groups (J-Synch 7 d: 3.0% and J-Synch 6 d: 5.0%). Consequently, calving rate was greater (P<0.05) in those recipients synchronized with the prolonged proestrus protocols (J-Synch 7 d: 35.6% and J-Synch 6 d: 36.9%) than with the short proestrus protocol (ECP: 29.7%).

**Table 1:** Estrus rate, utilization rate, pregnancy rates to embryo transfer (P/ET), pregnancy losses and calving rates in beef *Bos indicus* recipient cows synchronized with the J-Synch protocols (J-Synch 6 or J-Synch 7 d) and the conventional E2/P4 protocol (ECP).

Treatment groups	Estrus Rate	Utilization Rate	P/ET 30 d	P/ET 90 d	30 to 90-day Pregnancy losses	Calving Rate
J-Synch 7 d	82.0% (205/250) ^b^	40.0% (82/205)	37.0% (76/205)	7.0% ^a^ (6/82)	3.0% ^a^ (3/76)	35.6% ^a^ (73/205)
J-Synch 6 d	81.0% (203/250) ^b^	42.8% (87/203)	38.9% (79/203)	9.0% ^a^ (8/87)	5.0% ^a^ (4/79)	36.9% ^a^ (75/203)
ECP	89.0% (222/250) ^a^	41.8% (93/222)	35.1% (78/222)	16.1% ^b^ (15/93)	15.3% ^b^ 12/78	29.7% ^b^ (66/222)

ab Percentages within a column with different superscripts are different (P<0.05).

## GnRH treatment protocols

GnRH-based protocols have also been used to synchronize ovulation in recipients that received IVD (Baruselli et al., 2003; Hinshaw, 1999) or IVP (Ambrose et al., 1999; Sala et al., 2020) embryos. One of the main limitations for the application of protocols with GnRH in beef cows and heifers is the low response to the first dose of GnRH (Martinez et al., 2000) especially if they are *Bos indicus* (Batista et al., 2017; Silva et al., 2024). For this reason, a P4 device is inserted during the treatment to avoid early ovulations (Hinshaw, 1999). Furthermore, Sala et al. (2020) have shown that giving GnRH on day 0, is probably not necessary to synchronize Holstein heifers using the 5-day Cosynch+P4 protocol and receiving IVP embryos.

With the objective of increasing the response to the first GnRH treatment in beef cows. Bonacker et al. (2020a) developed a synchronization protocol called 7 & 7 Synch, using previous knowledge generated by Small et al. (2009). This protocol consists of applying PGF_2α_ and a P4 device on day -7 as a pre-synchronization treatment to develop a persistent follicle; on day 0, GnRH is administered to ovulate the persistent follicle and synchronize the emergence of a new follicular wave; on day 7, all cows receive PGF_2α_ and P4 device removal; and finally, all cows are FTAI with a dose of GnRH 60 to 66 h after device removal. The 7 & 7 Synch protocol demonstrated an improvement in the ovulatory response to the first GnRH administration (Bonacker et al., 2020a) and P/IA, both with conventional semen and with sexed semen, when compared against a 7-day Co-Synch+P4 treatment in suckled beef cows (Andersen et al., 2021). In addition, it was an interesting alternative in recipients when compared to the 7-day Co-Synch+P4 protocol, improving estrus expression (86.0% vs 76.3%), utilization rate (77.3% vs 70.35%) and pregnancy per synchronized recipient (40.3% vs 34.3%; P<0.05; Bonacker et al., 2020b).

Based on this knowledge and given the restrictions on the use of E2 in EU certified farms in Argentina and other countries, an alternative treatment based on GnRH was designed, which we called “Web-Synch” (Without Estradiol Benzoate). Briefly, this treatment is a slight modification of the 7 & 7 Synch (de la Mata et al., 2022). On day -5 a pre-synchronization treatment is initiated with the administration of PGF_2α_ and a P4 device to generate a persistent follicle. On day 0, GnRH is injected to induce ovulation of the persistent follicle and promote the emergence of a new follicular wave (36 h later). Subsequently, on day 6, the device is removed along with a dose of PGF_2α_ and eCG to promote final follicular growth and to induce a prolonged proestrus (as in the 5-day Co-Synch+P4 treatments), and tail paint is used for estrus detection. Finally, FTAI is performed 72-84 h after P4 device removal, with the application of GnRH only to animals that are not in estrus by that time. In most experiments carried out to evaluate the Web-Synch protocol in beef cows, P/IA were comparable to those obtained with E2/P4-based protocols (E2/P4: 49.8% (214/430) vs. Web-Synch 50.6% (214/430) in cows with a moderate to high incidence of cyclicity (40 to 50% of the cows with a CL on day 0; reviewed in Bó et al., 2022). However, in another group of cows in which only 9.8% of them had the CL on day 0, P/AI was greater in those receiving the conventional E2/P4 treatment (66.3%, 102/154) than those receiving the Web-Synch protocol (49.4%, 79/160; P=0.01). Although it was not evaluated in these studies, we speculate that differences may be due to a lower ovulation rate to the first GnRH in anestrus beef cows or to differences in the uterine environment due to lower E2 in the proestrus period in the cows not treated with ECP at P4 device removal.

However, results benefited the Web-Synch protocol in lactating dairy cows. The objective of the experiments that were recently conducted in lactating Holstein cows were to evaluate ovulatory follicle size, ovulation timing, and P/AI. Cows were synchronized with either the Web-Synch protocol or the conventional E2/P4-based protocol (named ECP, Macagno et al., 2022). In this case, the cows used were 160.0±7.1 d in milk, producing 35.6±0.8 kg of milk per day, with a 2.8±0.3 lactation period, with a BCS of 3.1±0.1, and were managed in a dry-lot system. Cows were randomly assigned to one of two treatment groups. On day 0, cows in the ECP group received 2 mg of EB (Estradiol, Over, Argentina) and an intravaginal device containing 1 g of P4 (Sincrover, Over). On day 6, cows received 150 µg D (+) cloprostenol (PGF_2α_, Prostal, Over). On day 7, the P4 devices were removed and cows received a second dose of PGF_2α_, 140 IU of reCG (FoliRec, CEVA), and 1 mg of ECP (Estrosinc, Over). Cows in the Web-Synch group were treated with PGF_2α_ and a P4 device on day -5 and 10 µg of buserelin (GnRH, Gestar, Over) on day 0. Removal of the P4 devices and treatment with PGF_2α_, and reCG was performed on day 6, and a second dose of PGF_2α_ was administered on day 7. Cows in both groups were painted at the base of their tail for estrus detection. In experiment 1, cows (n=39) were scanned twice daily from P4 device removal until ovulation. In experiment 2, cows (n=720) were treated similarly to those in experiment 1, but all those with >30% of their tail paint removed on day 9 (48 h after P4 device removal in the ECP group and 72 h after P4 device removal in the Web-Synch group) were AI at that time, and cows that without the tail paint removed in both groups received 10 µg of GnRH and were AI 12 h later. Cows in experiment 2 were also examined for pregnancy 30 d after AI. In experiment 1, the mean (±SEM) interval from P4 device removal to ovulation was longer (P<0.05) in the Web-Synch group (101.6±2.9 h) than in the control group (78.3±3.1 h), but the diameter of the ovulatory follicle did not differ (P=0.3; 19.7±0.8 and 18.5±0.8 mm for the Web-Synch and Control groups, respectively). In experiment 2, although no significant differences in estrus expression (P=0.3) were found between the Web-Synch (75.2%) and ECP (75.1%) groups, the P/AI was higher (P<0.01) in the Web-Synch group (51.5%, 183/355) than in the ECP group (41.3%, 151/365), respectively (Macagno et al., 2022). Furthermore, 66.5% of cows (236/355) ovulated at the first GnRH in the Web-Synch protocol and a greater proportion of cows had a CL at the time of PGF_2α_ in the Web-Synch (85.6%, 304/355) than in the ECP group (68,2%, 249/365; P<0,01). In summary, the GnRH-based synchronization protocol (Web-Synch) resulted in higher fertility than with the conventional E2/P4-based protocol in lactating dairy cows. The greater P/AI was possibly associated with a greater follicle wave synchronization after the GnRH administration compared to the EB treatment on day 0 and to a greater proportion of cows with a CL at the time of PGF_2α_ treatment in the Web-Synch than in the ECP group.

Considering the promising results obtained in lactating Holstein cows, an experiment was designed to evaluate P/ET, pregnancy losses and calving rates in lactating *Bos indicus x Bos taurus* recipient cows synchronized with the J-Synch or the Web-Synch protocol in Ecuador (Cedeño et al., 2024). Gyr x Jersey lactating cows (n=2131), 55.0±12.0 d in milk, a CL or at least one follicle >8 mm in diameter (determined by ultrasonography), 2.5 to 3.5 BCS and managed in a confinement system, were randomly allocated into one of two treatment groups. On day 0, cows in the J-Synch group (n=1125) received 2 mg EB (Calier, Ecuador) and an intravaginal device containing 1.2 g of P4 (Pluselar, Calier). On day 6, P4 devices were removed, and all cows received PGF_2α_ (Veteglan, Calier) and 400 IU eCG (Vetegon, Calier). Cows in the Web-Synch group (n=1006) were treated with PGF_2α_ and a P4 device on day -5, 10 µg buserelin (GnRH, Pluserelina, Calier) on day 0 and P4 device removal, PGF_2α_ and eCG on day 6. Cows in both groups were observed for signs estrus 72 h after device removal and those detected in estrus were examined by ultrasonography at the time of embryo transfer, 7 d after estrus (i.e. day 16). Recipients with a CL > 16 mm in diameter received Grade 1 IVP blastocyst by non-surgical transfer. Pregnancy was determined by ultrasonography at 30 and 90 d of gestation and were then followed until parturition to determine calving rates. Results are shown in Table 2. Recipients in the Web-Synch group had greater (P<0.05) P/ET and calving rates than those in the J-Synch group (Web-Synch: 42% and 36% vs J-Synch: 33% and 27.2%, respectively). Estrus and utilization rates, and pregnancy losses did not differ among groups (Table 2).

**Table 2:** Estrus rate, utilization rate, pregnancy rates to embryo transfer (P/ET), pregnancy losses and calving rates in Gyr x Jersey lactating recipient cows synchronized with the J-Synch or Web-Synch protocols.

Treatment groups	Estrus Rate	Utilization Rate	P/ET 30 d	P/ET 90 d	30 to 90-day Pregnancy losses	Calving Rate
Web-Synch	96.0% (1080/1125)	95.0% (1046/1125)	42.0% ^b^ (439/1046)	40.4% ^b^ (418/1046)	4.8% (21/439)	36.0% ^a^ (376/1046)
J-Synch	92.8% (934/1006)	91.0% (928/1006)	33.0% ^a^ (306/928)	31.0% ^a^ (288/928)	5.9% (18/306)	27.2% ^b^ (253/928)

ab Percentages within a column with different superscripts are different (P<0.05).

The results obtained in lactating cows were not confirmed in the heifers from the same farm (Bó et al., 2023). In this study, 2 year old Gyr x Jersey heifers (n= 375) with a CL (determined by ultrasonography), BCS between 3.5 and 4 and managed in a grazing system, were randomly allocated into the same treatment groups used in the previous experiment in lactating cows. The only difference was that in this case the heifers received a P4 device containing 0.6 g of P4 (Pluselar, Calier) and results are presented in Table 3. The utilization and P/ET were greater (P<0.01) and the pregnancy losses were lesser (P<0.01) in heifers in the J-Synch group compared to those in the Web-Synch group.

**Table 3:** Utilization rate, pregnancy rates to embryo transfer (P/ET), pregnancy losses and calving rates in Gyr x Jersey heifers synchronized with the J-Synch or Web-Synch protocols.

Treatment groups	Utilization Rate	P/ET 30 d	P/ET 90 d	Pregnancy Losses 30-90 d
Web-Synch	79.7% ^a^ (304/381)	33.8% ^a^ (103/304)	30.0% ^a^ (91/304)	11.6% ^a^ (12/103)
J-Synch	98.1% ^b^ (368/375)	51.0% ^b^ (188/368)	49.7% ^b^ (183/368)	2.1% ^b^ (4/188)

ab different superscripts indicate significant differences (P<0.01).

Similar results to the previous study in heifers were obtained in yet another study using Brahman, 2.5-year-old heifers with a CL (determined by ultrasonography), 3 to 4 BCS and managed in a grazing system (Pesantez et al., 2024). Heifers were randomly allocated into the same two treatment groups used in the previous experiment. In this case, selected heifers were also bled at the time of ET to determine plasma P4 concentrations and results are presented in Table 4. Although estrus and utilization rates did not differ among groups, heifers in the J-Synch group had greater (P<0.05) plasma P4 concentrations, 30 and 90-day P/ET and calving rates and lesser (P<0.05) 30 to 90-day pregnancy losses than those in the Web-Synch group. It was concluded that the J-Synch protocol is more appropriate than the Web-Synch protocol for the synchronization of *Bos indicus x Bos taurus* heifers used as embryo recipients.

**Table 4:** Estrus rate, utilization rate, pregnancy rates to embryo transfer (P/ET), pregnancy losses and calving rates in Brahman heifers synchronized with the J-Synch or Web-Synch protocols.

Treatment groups	Estrus rate	Utilization Rate	P4 ng/mL at ET	P/ET 30 d	P/ET 90 d	30 to 90-day pregnancy losses	Calving Rate
J-Synch	94.7% (109/115)	93.0% (107/115)	3.0±0.9 ^a^	47.0% ^a^(50/107)	44.8% ^a^ (48/107)	4.0% ^a^ (2/50)	41.0% ^a^ (44/107)
Web-Synch	88.7% (102/115)	87.0% (100/115)	2.1± 0.5 ^b^	39.0% ^b^(39/100)	32.0% ^b^(32/100)	18.0% ^b^ (7/39)	25.0% ^b^ (25/100)

ab different superscripts indicate significant differences (P<0.05).

The divergent results between lactating cows and heifers in the FTET and between lactating dairy cows and beef cows in FTAI programs are interesting to analyze. Although it was not evaluated in all these studies, we speculate that differences may be due to a lower ovulation rate to the first GnRH in anestrus beef cows and in the cycling heifers than in the lactating dairy cows. Therefore, further improvements are needed in the Web-Synch protocol for the synchronization of beef cows or heifers.

## Summary and final conclusions

The protocols developed for FTET over the last 20 years have provided practitioners with the greatest opportunity for transferring large number of embryos in recipient herds and has been pivotal for the development of the large scale IVP embryo-derived industry in South America. Although overall P/ET have been considered adequate for most practitioners, there are factors such as the expression of estrus and pregnancy losses that need to be considered for successful embryo transfer programs. The present manuscript has reviewed several studies showing a positive correlation between the manifestation of estrus, P/ET and pregnancy maintenance in recipients. Furthermore, the implementation of protocols with prolonged proestrus, like the J-Synch, have appeared as an interesting alternative for heifers and beef *Bos indicus* recipient cows. Finally, the long GnRH-based protocols, like the Web-Synch presented herein, seem to be an interesting alternative for lactating dairy cows, but further improvements are needed in order to implement this protocol in heifers.

## Data Availability

Research data is available in the body of the article.
